# Osteoid Osteoma in an Adult Wheelchair Basketball Player Mimicking Musculoskeletal Shoulder Pain: Red Flag or a Red Herring?

**DOI:** 10.3390/tomography8010032

**Published:** 2022-02-07

**Authors:** Filippo Maselli, Lorenzo Storari, Mariangela Lorusso, Firas Mourad, Denis Pennella, Valerio Barbari, Mattia Salomon, Fabrizio Brindisino

**Affiliations:** 1 Sovrintendenza Sanitaria Regionale Puglia INAIL, 70126 Bari, Italy; masellifilippo76@gmail.com; 2 Department of Neurosciences, Rehabilitation, Ophthalmology, Genetic and Maternal Infantile Sciences (DINOGMI), Campus of Savona, University of Genova, 17100 Savona, Italy; valeriobarbari1993@gmail.com; 3 Department of Clinical Science and Translation Medicine, Faculty of Medicine and Surgery, University of Rome Tor Vergata, 00133 Roma, Italy; mariangela.lorusso@hotmail.it (M.L.); denispennella@gmail.com (D.P.); salomon.mattia@gmail.com (M.S.); fabrizio.brindisino@unimol.it (F.B.); 4 Department of Physiotherapy, LUNEX International University of Health, Exercise and Sports, 4671 Differdange, Luxembourg; firas.mourad@me.com; 5 Luxembourg Health & Sport Sciences Research Institute A.s.b.l., 50, Avenue du Parc des Sports, 4671 Differdange, Luxembourg; 6 Department of Medicine and Health Science “Vincenzo Tiberio”, University of Molise C/da Tappino c/o Cardarelli Hospital, 86100 Campobasso, Italy

**Keywords:** differential diagnosis, osteoma osteoid, neoplasms, bone tissue, rotator cuff tendinopathy, physical therapists

## Abstract

Osteoid osteoma (OO) is a relatively common, benign bone-forming tumour, which mainly occurs on the long tubular bones of the limbs in adolescents. Usually, the OO is classified based on its localisation. Night-time pain is the major symptom of OO, which is commonly relieved using non-steroidal anti-inflammatory drugs, while surgery is required only for those patients with severe pain or in case of failure of previous conservative treatments. Our case report describes a 56-year-old male basketball player who self-referred to our outpatient physical therapy with a shoulder pain complaint. Considering the anamnesis and the physical examination, the physical therapist referred the patient to an orthopaedic surgeon, who suggested a detailed imaging investigation. The peculiarity of this clinical case is the overlapping of two clinical presentations: the symptomatology of the OO and the concurrent mechanical disorder due to a rotator cuff tendinopathy.

## 1. Introduction

Shoulder pain (SP) is the third most common musculoskeletal disorder (MSD) after low back pain and neck pain [1,2], and one of the most prevalent complaints in outpatient physical therapy clinics [1,2]; whereas it represents the third most frequent cause of consultation in the emergency department [3,4]. Most diagnoses of SP provided by healthcare professionals are impingement syndrome, rotator cuff tendinopathy, and adhesive capsulitis [3,4]. Furthermore, SP makes up a large proportion of MSDs for disabled people using wheelchairs, which are commonly caused by the increased load and the repetitive stress of wheelchair handling [5,6]. The risks to develop SP increase when practising wheelchair basketball (WB), which is the most popular sport for individuals with lower-leg motor impairments [7,8,9]. In previous studies, approximately 72–85% of WB players reported SP related to daily life or sports activities [10]. This is probably due to specific playing movements of WB, such as pushing or turning the wheelchair, and overhead movements, such as basket shooting or bouncing, which in the long term may play a role in SP [11,12,13]. Fortunately, most shoulder complaints are attributable to non-specific MSDs, but in some cases, healthcare professionals (HPs) should also consider an underlying visceral or severe condition among pathologies that could manifest as SP, such as tumours, cancer, and pulmonary and cardiovascular pathologies [14,15]. Among these conditions, osteoid osteoma (OO) is a relatively common tumour, usually non-aggressive and not susceptible to malignant changes, and it accounts for 10–12% of benign bone tumours in adulthood [16,17,18,19]. About 70% of OO usually develops in patients younger than 20 years [18]; however, it may also occur in the mature skeleton up to the age of 70 years [18]. Although OO was first described in the 1930s [20], its aetiology is still unknown [16,21]. Statistically, men have three times more of a chance than women to be affected [22,23], and this benign tumour occurs mainly on the bones of the appendicular skeleton. Lower limb bones are the most affected, roughly in more than 80% of the cases, while upper limbs are less frequently involved (i.e., 19–30%). Notably, the femur and tibia are affected in 80% of cases, ref. [19] whereas hands and feet are affected in 30% [24,25]. Conversely, some atypical locations reported for OO are the skull, ribs, ischium, mandible, patella, proximal humerus, and scapula [24,25,26]. The size of OO is usually small, measuring 1.0–2.0 cm in width, and it is classified into different subtypes based on its localisation within the cortical, spongious, or sub-periosteal zone [19,21]. Radiographically, OO is characterised by a central nucleus called “nidus”, a highly vascularised and innervated structure surrounded by a sclerotic bone area [19,21,26,27,28], in which the presence of several prostaglandins determines chronic reactive processes and increased intra-cortical pressure [29,30], causing the typical OO-related pain. This latter is usually well localised; it worsens during the night, and it is relieved only by the intake of non-steroidal anti-inflammatory drugs (NSAIDs) [19], while swelling is the second most referred to symptom [31].

As with other bone tumours, common plain radiographs represent first-line examination [19]; however, in the case of unremarkable findings, the second-line diagnostic options are three-phase bone scans [22] and computed tomography (CT), which is the gold standard for the diagnosis and localisation (cortical, sub-periostal, and medullary) of OO [19,21]. Conversely, even if the magnetic resonance imaging (MRI) is more sensitive than CT in detecting reactive changes of soft tissues, the bone marrow oedema seen with MRI may conceal typical characteristics of the OO, such as the “nidus”; therefore, the latter is less useful than CT for diagnostic purposes [19,21]. Clinically, manifestation patterns of OO can be misdiagnosed as a common MSD, challenging the HPs’ diagnosis process [28,32,33,34,35]. For instance, when OO is located in the shoulder, it may mimic an impingement syndrome and SP [28,32,33,34].

The conservative management of symptomatic OO takes about 33 months [31], with major side effects related to prolonged use of NSAIDs [12]. However, surgical treatment should be considered only for those patients with severe pain and who did not respond to conservative treatment [21]. Furthermore, it is also reliable for those patients who are unwilling to tolerate pain and for those at risk of kidney or gastrointestinal complications due to prolonged NSAIDs intake [21]. Surgical approaches may require large bone resection, block resection, graft transposition, arthrotomy, or joint dislocation; otherwise, the percutaneous CT-guided surgical approach only requires a small access [35,36].

The aim of this article is to describe the clinical presentation and physical examination of a WB athlete with SP masked as OO. It also describes the clinical reasoning of a physiotherapist (PT) who works in a direct access setting, which led to the consultation of another HP to deepen the diagnosis of the patient and to promote the best clinical management.

## 2. Case Presentation

**Figure 1:**
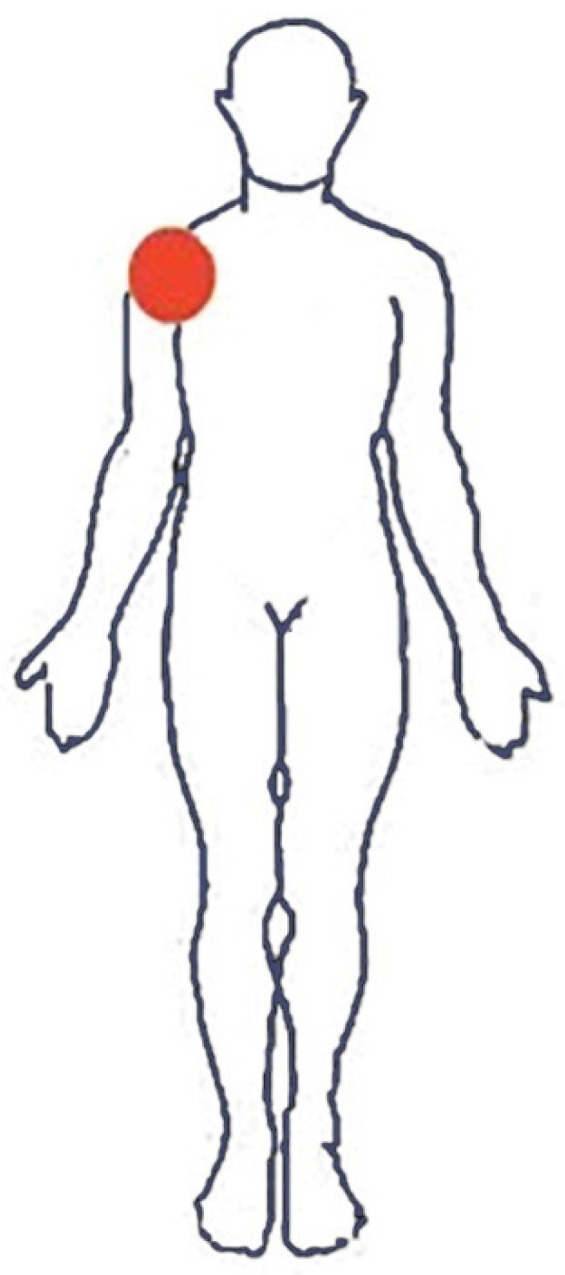
Body chart at pain onset. Bright red indicates the initial location of the painful body area.

**Figure 2:**
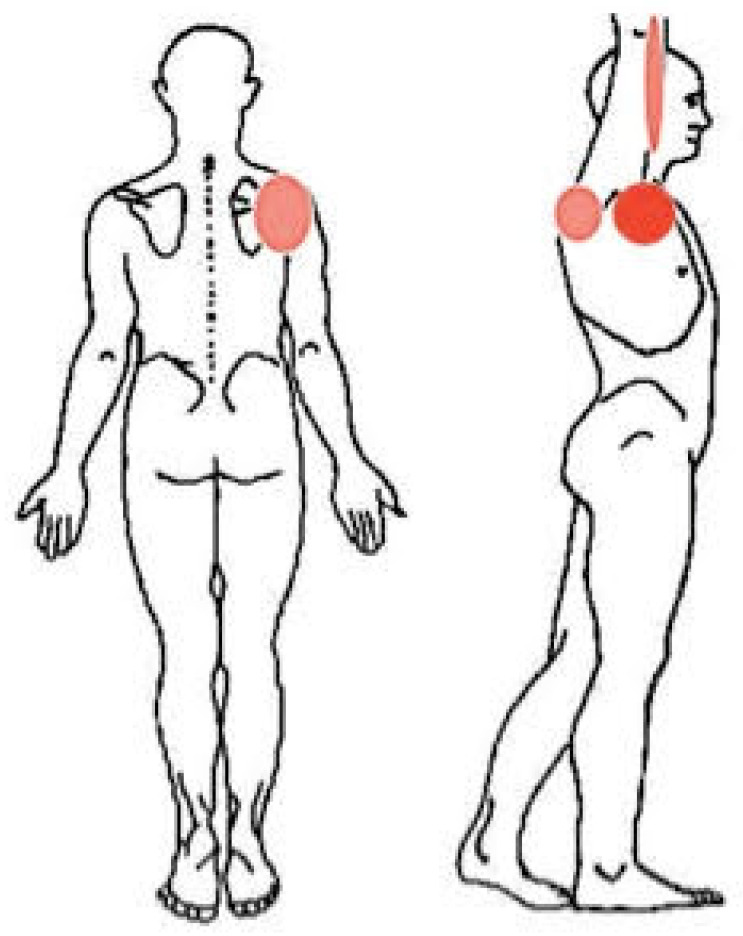
Body chart after 1 month. Bright red indicates the most painful body areas; pale red indicates the mildly painful body areas.

A 56-year-old WB player and employee self-referred to our outpatient physical therapy clinic complaining of right SP. He had been playing WB for 15 years at a professional level (i.e., Italian B-league WB championship). The patient suffered from poliomyelitis since early childhood, which forced him into a wheelchair. He reported that SP gradually started in the last 2 months, without any trauma or training changes. In the beginning, he continued to work and play WB regularly because SP was bearable. However, after 1 month, it worsened while performing a high-resistance overhead pull-down exercise during a WB training session. The patient referred to an acute pain on his anterior right shoulder, rated as a 5/10 on the numeric pain rating scale (NPRS) [37] (Figure 1). During the following 2 weeks, SP increased to a 6/10 NPRS due to WB, walking with crutches, and performing daily living activities requiring flexion or abduction of the shoulder above 50° or cross-body adduction movements. Thus, the pain became constant and deep. In addition, there were no soothing movements or positions. Interestingly, over time, pain further worsened during the night (7/10 NPRS), especially in a supine position, forcing the patient to take NSAIDs (i.e., ibuprofen, 400 mg) every night to improve his sleep, with a partial decrease in symptoms. Lately, he also noticed a new superficial pain associated with tingling on the backside of his shoulder, which radiated to the medial part of his arm (3/10 NPRS) (Figure 2). In his past medical history, the patient reported two previous surgeries because of a right Achilles tendon tear and denied any systemic symptoms such as fever, unexplained recent weight loss or gain, and any bowel or bladder symptoms. Notably, the patient is a blood donor with a frequency of four times a year, and his last blood tests were unremarkable. Moreover, in the last 2 months, no infections or travel abroad were reported. Because of these symptoms, he was forced to use the wheelchair instead of crutches and decided to contact his general practitioner (GP). The GP, after a brief evaluation based mainly on the anamnesis, made a diagnosis of subacromial impingement syndrome (SIS), prescribed him pain killer drugs (i.e., Tramadol), and referred him to a physiotherapy service for 10 sessions of laser therapy and exercises. For this reason, the patient decided to seek his PT for further evaluation.

### Investigations

At the first visit at the physiotherapy outpatient clinic, the patient presented with his wheelchair because of SP, and thus the physical examination was performed in a sitting position. No deformities in the right shoulder were noted during observation. The patient localised the SP specifically to the anterior aspect of the right shoulder, just below the acromioclavicular joint, and reported a moderate (6/10 NPRS) and constant pain. The palpation of the coracoid bone reproduced the patient’s own pain. An active movement assessment by wireless inclinometer (Tracker Freedom^®^ JTECH Medical, Midavele, UT, USA) was performed, revealing a decreased active range of motion (ROM) of the right shoulder during anterior elevation (150°/180° right shoulder compared to 180°/180° left shoulder) and in abduction (160°/180° right shoulder compared to 180°/180° left shoulder). All active movements were painful (7/10 NPRS), whereas passive ROM evaluations were pain-free, except for passive horizontal cross-body adduction movements with overpressure, which reproduced the patient’s own pain (7/10 NPRS). To evaluate the symptoms of SIS, the PT also performed a set of orthopaedic shoulder tests: the Neer sign, painful arc sign, Hawkins–Kennedy test, the internal rotation resistance strength test, and the Yocum test, but none of them reproduced the patient’s own pain. However, literature findings highlight these tests for specific soft tissue injury of the shoulder as unreliable manoeuvres to detect the onset of pain and often are only useful as pain-provocation manoeuvres within the clinical reasoning [38,39,40,41,42]. Then, the cervical spine was assessed to frame the tingling and radiating pain to the arm: active and passive ROM was pain-free and normal; no evidence of tenderness or muscle spasm. The Spurling test for foraminal compression [43] and the neck distraction test [43] were both negative; furthermore, the upper limb neurodynamic test one, performed to evaluate a neural tissue involvement, was negative. Finally, the peripheral neurological examination (i.e., sensory, motor, and deep tendon reflexes testing) of the upper quadrant was unremarkable. In addition, a set of provocation tests in specific loading activities was performed; resisted shoulder elevation at 90° and resisted external rotation reproduced the patient’s primary complaint (i.e., anterior pain below the acromioclavicular joint). A shoulder disability questionnaire (SDQ) [44] to assess shoulder related activity, participation, and psychosocial factors and Short Form 36 (SF-36) [45] to evaluate the quality of life were also administered. Accordingly, although not completely fitting with the clinical manifestation, the PT hypothesised that the patient’s clinical presentation was suggestive of a rotator cuff tendinopathy [46], and therefore started a physiotherapy program based on education, manual therapy techniques, desensitisation manoeuvres, and gradual re-load exposure [47,48,49]. However, based on history-taking (i.e., age, insidious onset of pain due to overload activities/training, pain intensity and persistence, night pain enhanced in a supine position, and NSAIDs abuse) and clinical (i.e., familiar pain evoked by coracoid compression) findings, the PT also referred the patient to an orthopaedic surgeon for a detailed imaging examination, aiming to screen any potential non-musculoskeletal sources of pain (i.e., a treat and refer regimen) [50,51,52,53].

## 3. Results

### 3.1. Differential Diagnosis

After a detailed history-taking and clinical examination, the orthopaedic surgeon agreed with the PT about the possible involvement of non-musculoskeletal pain in addition to the rotator cuff tendinopathy and prescribed shoulder pain radiographs and MRI.

### 3.2. Imaging

**Figure 3:**
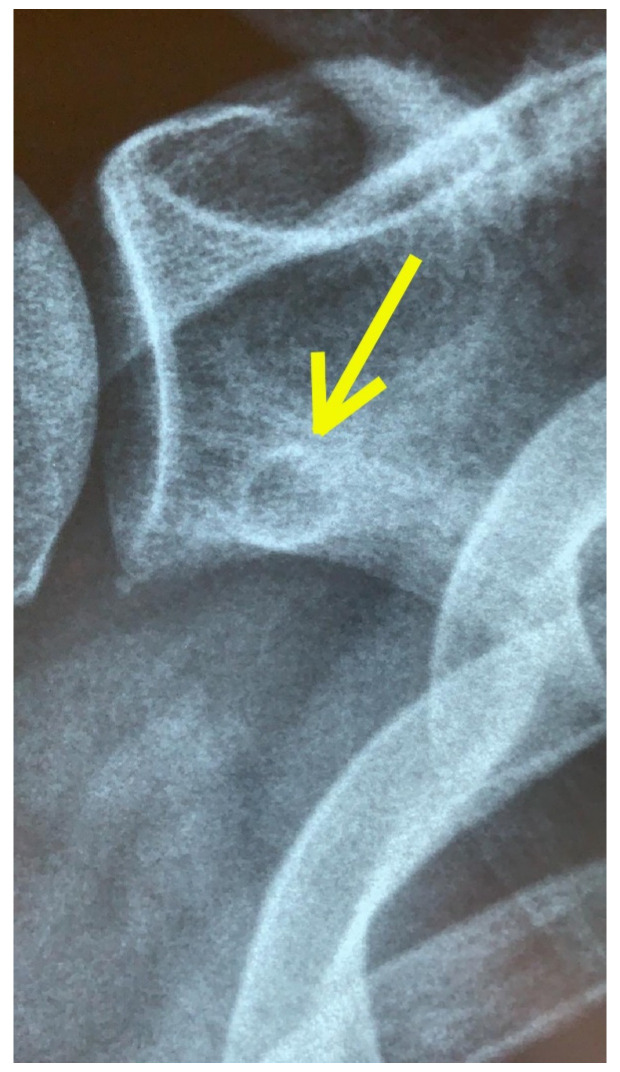
X-ray imaging. A small radiolucent formation is present on the lower portion of the scapular neck with hyperdense margins and an intact cortical rim of non-univocal interpretation (yellow arrow).

**Figure 4:**
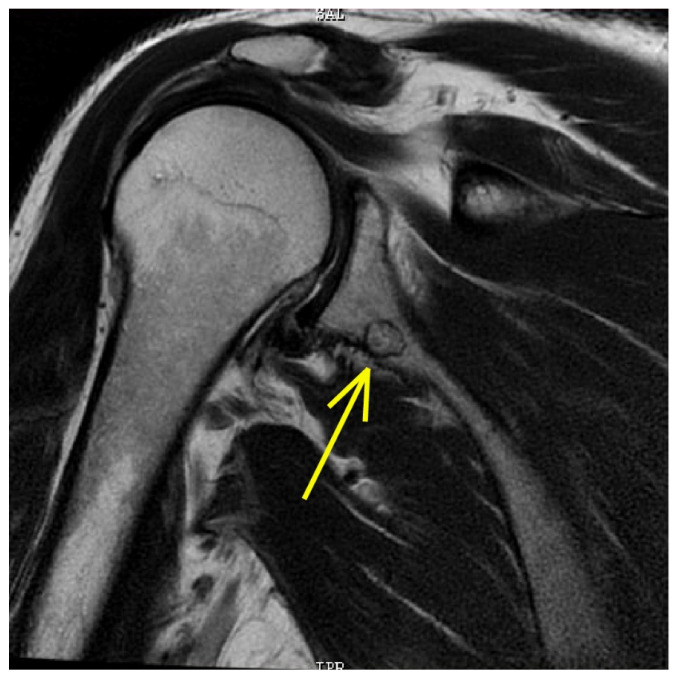
MRI scan. A roundish centimetric formation with a surrounding osteosclerotic border is visible at the lower portion of the scapular neck. This lesion has an uneven signal due to the presence of a central component with intermediate signal intensity (nidus) and is compatible in the first instance with osteoid osteoma with an atypical site (yellow arrow).

The plain radiographs (Figure 3) showed a “small radiolucent formation at the lower portion of the scapular neck with hyperdense margins, and an intact cortical rim of uncertain interpretation”, while the MRI clearly described the presence of a “roundish centimetric formation with surrounding osteosclerotic border in the lower portion of the scapular neck. This lesion showed a central component with intermediate signal intensity (nidus) and is compatible in the first instance with OO with atypical localization” (Figure 4). Moreover, MRI reported a tendinosis of the subscapularis muscle, minimal tenosynovitis of the long head of the biceps tendon, and fibroadipose degeneration of the teres minor muscle.

### 3.3. Treatment

The orthopaedic surgeon diagnosed the OO. Through a shared decision-making process involving the patient, conservative management was suggested (i.e., physiotherapy and acetylsalicylic acid drug intake for pain control).

Periodic controls every 2 months were planned to evaluate any evolution of the OO. The PT performed 12 sessions (i.e., 3 sessions during the first 2 weeks, 2 sessions per week during the following 2 weeks and a single session during the last two weeks) consisting of: pain neuroscience education [54], graded activities exposure both during sports and home management, and manual therapy for cervical–thoracic junction and the right shoulder [49]. Finally, strengthening exercises and load management to reduce and to retrain shoulder function were performed in adjunct to proprioceptive and specific sport exercises focusing on overhead activities. Table 1 summarises the rehabilitation progression. 

### 3.4. Outcome and Follow-Up

The outcomes were collected using patient-reported questionnaires submitted to the patient during the first visit and at the 6th week follow-up. At the 6th week, pain intensity was completely resolved to 0/10 NPRS; significant improvements were also observed on SDQ and SF-36 scores. Detailed results are provided in Table 2.

**Figure 5:**
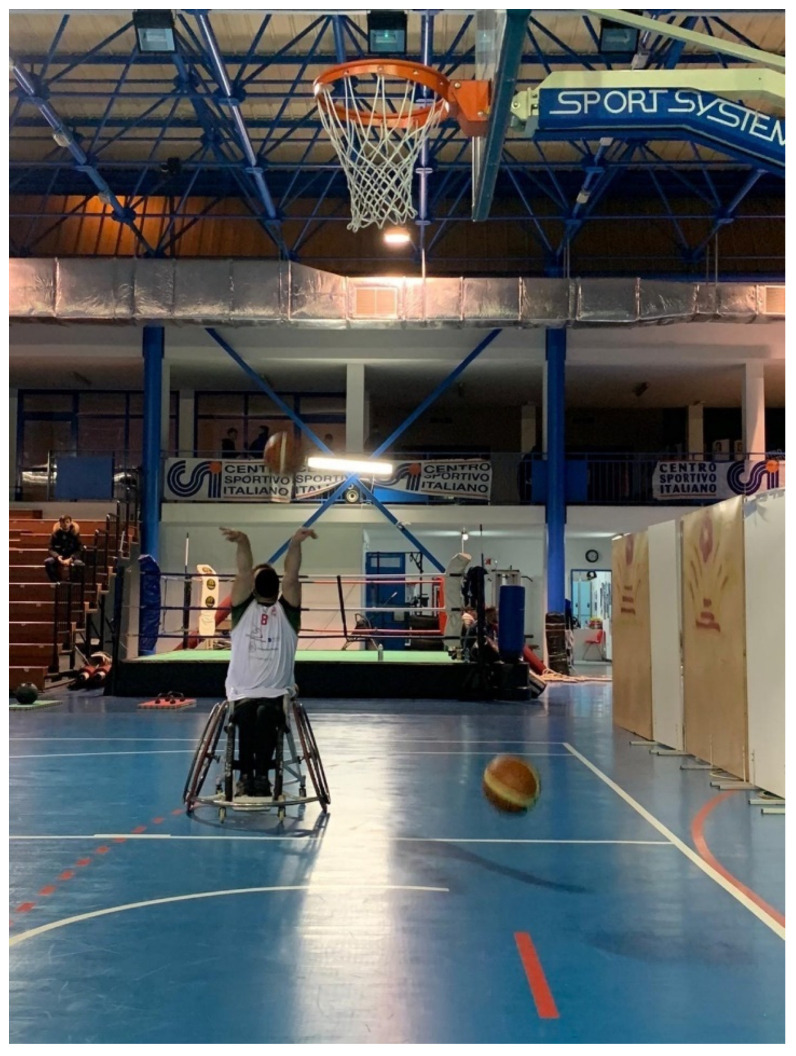
Wheelchair basketball. Picture of the patient in-wheelchair during a training session.

Moreover, passive and active ROM were pain-free, as well as the cross-body adduction movement, which initially reproduced the most painful symptom. Furthermore, the patient reported the absence of anterior SP at rest and a pain-free return to sports activities (Figure 5). Notably, plain radiographs and MRI scans performed on the 6th week showed no changes of the OO compared to the baseline imaging.

## 4. Discussion

Our case report highlights that OO may clinically mimic, otherwise being concurrent to a common musculoskeletal condition such as SP [19,55]. Data gained from medical history, clinical examination, and diagnostic imaging are fundamental for appropriate HP management, which needs a careful triage and early diagnosis to decide if the patient is suitable for rehabilitation or surgery. In our case, the patient presented the clinical characteristics of shoulder tendinopathy; however, the awareness of the PT to all features of the physical examination led to suspecting the concomitant presence of a concealed pathology beyond his scope of practice. Notably, this case is also remarkable because the scapula is an unusual location for OO [15,16,56,57,58]. For this reason, it is essential for every HP to be aware of the clinical meaning of particular and atypical clinical manifestations of pathologies, which could be outside of the proper scope of practice. In this regard, all HPs should collaborate with each other to reduce the timing of the diagnostic processes and plan the best path of care. The diagnostic process often needs a teamwork approach to evaluate all the possible scenarios, in which each professional knows how to fulfil his role. In Italy, this is fundamental, especially when the patient needs detailed imaging, and prescriptions are a specific relevance of GP and medical doctors. Indeed, a radiological investigation is often needed to assess the shoulder region. In this case, the findings of the X-ray scans and the presence of high-signal intensity on T2-weighted MRI images with a well-defined ‘‘nidus’’ confirmed the suspicion of OO [55,59]. The HPs must always be conscious that patients might show a clinical presentation not completely suitable with the main features of a specific pathology. In fact, many serious pathologies (e.g., Brodie abscess, stress fracture, bone island, eosinophilic granuloma, and malignant tumours such as Ewing sarcoma and osteosarcoma) may affect the bone tissue, [20,25,60,61]. With regard to SP, several extra expertise pathologies have been shown to cause SP [62,63,64]; however, only a few of them have signs and symptoms sufficiently informative to raise the suspicion of such medical pathology.

Accordingly, in our case report, the clinical and the radiological findings should not be considered as red flags but red herrings, which are useful to track the evolution of the clinical conditions in terms of disability or persistence of symptoms. This is in line with the current literature regarding the screening for referral processes [48,50,52,53]. HPs must be aware of the low diagnostic accuracy—and then of the clinical reliability—of several red flags commonly used in MSD management; thus, they need to base their clinical reasoning on careful history-taking, risk factors analysis, and objective physical examinations (e.g., the review-of-systems) [65,66].

Notably, the peculiarity of this case relies on the patient’s musculoskeletal signs and symptoms, such as SP—which is very common in overhead athletes, especially in WB players [67,68] —and the concomitant symptomatic OO in the same anatomic region. The choice of the patient to avoid the surgery (due to the possible impact on his shoulder function and his walking ability with crutches) highlighted the low weight of the structural pathology—the OO—over the major cause of symptoms, which was a rotator cuff tendinopathy. In fact, OO is a benign skeletal tumour, and its clinical and radiological presentation may be unclear and even asymptomatic [12]. Interestingly, it is difficult to define the role of OO and its involvement as a cause of SP. In fact, despite good responses to pharmacological therapy and rehabilitation, at the 6-month follow-up, the imaging showed no significant changes of the OO compared to the baseline assessment. However, our experience highlights that the PT must always consider any potential clinical scenario of the underlying causes. When history-taking and clinical presentation show warning signs or symptoms—such as painful manual striking on the bone tuberosity—the patient may be suitable for a further examination [69]. Notably, clinicians must be aware also that anatomical findings on imaging investigations could be asymptomatic [70].

In fact, as in this case, the imaging findings did not influence the outcomes of the conservative management that led to a pain-free return to play. This underlines, once again, that those findings can be asymptomatic and that association between symptoms and current pathology is still poorly understood [71]. Accordingly, many authors proposed the use of the term non-specific SP following the current literature on lower back pain and neck pain [71,72,73,74]. Finally, this case report highlights the importance of a multidisciplinary healthcare teamwork approach during the differential diagnosis process of a rare presentation of a common disease and the importance of a shared decision-making process involving both patient and HP expertise to realise a tailored path of care [74].

## 5. Conclusions

The role of a PT is crucial for an appropriate evaluation and management of a patient complaining of an MSD, particularly if a proper medical referral is needed. In this regard, an exhaustive screening for referral is a mandatory step for every HP, especially for those in a direct access setting. Notably, certain medical conditions, such as OO, may be a concurrent manifestation of other most-common MSDs, such as SP. Advanced clinical reasoning skills may drastically change patient prognosis and reduce the risk of a misdiagnosis. Finally, the radiological findings of pathologies requiring surgical treatment should not exclude a conservative approach that could represent the first-line choice for certain patients, especially when symptoms are not clearly related to imaging findings.

## Figures and Tables

**Table 1:** Rehabilitation treatment.

**1st week (3 sessions)**	➢ Patient education for the management of pain and progressive load during the execution of ADL and sports activity; ➢ Manual therapy, in the specific treatment of the trigger points of the right shoulder and cervico-dorsal muscles; ➢ Initial exercises to strengthen the upper limb and the shoulder–thoracic girdle to reduce pain and restore function.
**2nd week (3 sessions)**	➢ Patient education regarding progress achieved during the first week; ➢ Manual therapy, in the specific treatment of the trigger points of the right shoulder and cervico-dorsal muscles; ➢ Progressive strengthening exercises of the upper limb and the shoulder–thoracic girdle for pain reduction and recovery of function.
**3rd week (2 sessions)**	➢ Patient education regarding progress achieved during the previous weeks; ➢ Manual therapy, in the specific treatment of the trigger points of the right shoulder and cervico-dorsal muscles; ➢ Moderate and progressive strengthening exercises of the upper limb and the shoulder–thoracic girdle for pain reduction and recovery of function.
**4th week (2 sessions)**	➢ Patient education regarding progress achieved during the previous weeks; ➢ Progressive strengthening exercises of the upper limb and the shoulder girdle for the reduction in pain and the recovery of sporting function and gesture; progressive sport-specific exercises, proprioceptive exercises, and exercises focused on overhead activities have been added.
**5th week (1 session) and 6th week (1 session)**	➢ Patient education regarding progress achieved during the previous weeks; ➢ Progressive strengthening and proprioception exercises of the upper limb and the scapulo-thoracic shoulder girdle, more focused on recovery and optimisation of the sporting gesture.

**Table 2:** Outcomes measurement.

**1st Day Examination**								**6th-Week Follow Up**							
**NPRS 6/10**								**NPRS 0/10**							
**SDQ 12 (activity)** **8 (participation, psychosocial factors)**								**SDQ 2 (activity)** **1 (participation, psychosocial factors)**							
**SF 36**								**SF 36**							
**Physical Functioning**	**Limitations—Physical Health**	**Pain**	**General Health**	**Energy/Fatigue**	**Social Activities**	**Limitation—Emotional Problems**	**Emotional Well-Being**	**Physical Functioning**	**Limitations—Physical Health**	**Pain**	**General Health**	**Energy/Fatigue**	**Social Activities**	**Limitation—Emotional Problems**	**Emotional Well-Being**
**35**	**0**	**30**	**37**	**55**	**25**	**66**	**56**	**70**	**100**	**61**	**72**	**75**	**75**	**100**	**80**

## Data Availability

Not applicable.
